# Toward an ecological analysis of Bayesian inferences: how task characteristics influence responses

**DOI:** 10.3389/fpsyg.2015.00939

**Published:** 2015-08-04

**Authors:** Sebastian Hafenbrädl, Ulrich Hoffrage

**Affiliations:** Faculty of Business and Economics, University of LausanneLausanne, Switzerland

**Keywords:** Bayesian inference, updating beliefs, ecological analysis, task characteristics, base rate, signal-detection, representation format, natural frequencies

## Abstract

In research on Bayesian inferences, the specific tasks, with their narratives and characteristics, are typically seen as exchangeable vehicles that merely transport the structure of the problem to research participants. In the present paper, we explore whether, and possibly how, task characteristics that are usually ignored influence participants’ responses in these tasks. We focus on both quantitative dimensions of the tasks, such as their base rates, hit rates, and false-alarm rates, as well as qualitative characteristics, such as whether the task involves a norm violation or not, whether the stakes are high or low, and whether the focus is on the individual case or on the numbers. Using a data set of 19 different tasks presented to 500 different participants who provided a total of 1,773 responses, we analyze these responses in two ways: first, on the level of the numerical estimates themselves, and second, on the level of various response strategies, Bayesian and non-Bayesian, that might have produced the estimates. We identified various contingencies, and most of the task characteristics had an influence on participants’ responses. Typically, this influence has been stronger when the numerical information in the tasks was presented in terms of probabilities or percentages, compared to natural frequencies – and this effect cannot be fully explained by a higher proportion of Bayesian responses when natural frequencies were used. One characteristic that did not seem to influence participants’ response strategy was the numerical value of the Bayesian solution itself. Our exploratory study is a first step toward an ecological analysis of Bayesian inferences, and highlights new avenues for future research.

## Introduction

A woman receives a positive HIV test—what is the probability that she is infected? An eyewitness claims that she saw a blue cab involved in an accident—what is the probability that the cab was actually blue? A potential customer asks for a second sales presentation—what is the probability that he will ultimately place an order? Even though these questions come from different domains, they all share the same underlying structure: an individual receives new diagnostic information and wants to update her beliefs accordingly. Tasks that provide (a) information about prior probabilities of some hypotheses, (b) information that new evidence is available, and (c) information about the probabilities of such new evidence under various conditions, are called Bayesian inference problems and their solution can be calculated using Bayes’ rule. For more than 50 years, researchers have been interested in the psychological processes individuals deploy to solve such problems as well as how to help individuals to solve such problems more effectively (Mandel, 2014, 2015; Johnson and Tubau, 2015; McNair, 2015).

Sirota et al. (2015) pointed out that Bayesian reasoning is not restricted to textbook problems, and that there is a wide range of situations that call for Bayesian reasoning “in the wild,” that is, in real life contexts in which information is usually *not* provided in numerical form and in which people (and animals) nevertheless have to behave after some events occurred or after some information became known (see also Griffiths and Tenenbaum, 2006). This distinction is akin of Hertwig et al.’s (2004) distinction between *decisions-from-description* and *decisions-from-experience*. In a similar vein, Mandel (2014) called for adopting a wider perspective and for studying Bayesian reasoning in domains other than textbook problems. In the present paper, we do not follow this call, and we analyze, as most researchers on Bayesian reasoning do, people’s responses to textbook problems. Yet, we aim at going beyond the usual treatment of such problems. Usually, the content of a given task is just regarded as decoration—what is important is that the task has a certain structure and that this structure and the information given in the task qualify it as a Bayesian inference task for which Bayes’ rule, as a “content-blind” norm, provides the solution (Gigerenzer, 1996). We question what often seems to be taken for granted, namely that task content does not matter and is exchangeable. This avenue does not lead us into the wild, but it leads us into white territory from the viewpoint of classic textbook problem analysis. We seek to explore the effect of task dimensions that are usually ignored.

We are not the first to challenge the tacit assumption that task content is decorative and can be ignored as long as it serves its purpose, namely to convey what the structure is and which normative principle applies. For instance, Cosmides (1989) argued that the content and context of the task used to study deductive reasoning matters: while a Wason selection task with an abstract content yields very few normatively correct responses, people’s ability to correctly apply the modus tolens increased dramatically when the rule that needed to be checked was formulated as a social contract—even though this was irrelevant from a logical point of view. Another example is Krynski and Tenenbaum’s (2007) finding that performance in a Bayesian reasoning task depends on verbal content, specifically, whether a reason for a false-alarm in a medical test has been given (“the presence of a benign cyste”) or not. Note that providing participants with an alternative cause for a positive test is irrelevant from a normative point of view because it does not affect the false-alarm rate. In other words, the false-alarm rates in both versions, with and without reason for the false-alarm, were the same. However, providing a reason boosted the proportion of Bayesian answers from about 25% to about 45%—which is, according to Johnson and Tubau (2015), “some of the highest performance reported with normalized data in the absence of visual cues.” To provide one more example, Mellers and McGraw (1999) hypothesized that the beneficial effect of natural frequencies (for an explanation of this concept, see below) is minimized for tasks with a high base rate, which amounts to saying that the usage of a Bayesian response strategy in the probability/percentage version and in the natural frequency version is differentially affected by the base rate stated in the problem. Note that the claim is not that the Bayesian solution depends on the base rate—this is trivial and follows from Bayes’ rule. Rather the claim is empirical in nature, namely that a participant’s chance of answering with the Bayesian solution does depend on the base rate. Mellers and McGraw (1999) provided supportive evidence for their interaction hypothesis, and when we tested it with our own data, we could confirm that the pattern of results for the cab problem (which Mellers and McGraw used) seemed indeed to be special, but we could not obtain supportive evidence for the hypothesized interaction in general (Gigerenzer and Hoffrage, 1999).

Our research question is directly in line with these three examples: are there characteristics of Bayesian textbook problems—and if so, which—that influence participants’ responses? Note that this investigation conceives participants’ responses to Bayesian inference tasks as a function of task characteristics and can thus be considered as an example of how strategy usage depends on ecological dimensions (Todd et al., 2012).

## Materials and Methods

### Databasis

To explore how characteristics of Bayesian inference tasks influence responses and the usage of response strategies, we reanalyzed data that was obtained by prior research. In particular, we pooled the data from Hoffrage et al. (2015) and the data from Study 1 of Gigerenzer and Hoffrage (1995). Our pooled data set consists of 19 different tasks (4 tasks from Hoffrage et al., 2015 and 15 tasks from Gigerenzer and Hoffrage, 1995), presented to a total of 500 different participants who provided 1,773 responses. **Table 1** gives an overview of these tasks and how they score on various quantitative and qualitative dimensions (which will be introduced in more detail below).

**Table 1:** The 19 tasks used in the present analysis, ordered according to base rates.

	Task Content		Quantitative Dimensions					Qualitative Dimensions			
Task	Hypothesis (H)	Data (D)	Base rate	Hit rate	False-alarm	Size of Reference	Bayesian	Norm	Stakes	Main	Number of
			(Br)	(Hr)	rate (F)	Class	response (Bay)	deviation (N)	(S)	focus (M)	responses
1	Pimp	Wearing a Rolex	0.005	80	0.05	1000000	7.41				60
2	HIV infection	HIV-test positive	0.01	100	0.1	1000000	9.09	1	1	1	60
3	Heroin addict	Fresh needle prick	0.01	100	0.1	100000	5	1	1	1	60
4	Committing suicide	Professor	0.024	15	12	1000000	0.03	1	1		60
5	Prenatal damage in child	German measles in mother	0.21	47.6	0.5	10000	16.7	1	1	1	60
6	Breast cancer	Mammography positive	1	80	9.6	1000	7.77	1	1	1	60
7	Car accident	Driver drunk	1	55	5.05	10000	9.91	1	1		60
8	Pregnant	Pregnancy test positive	2	95	0.51	1000	79.17	0.5	1	1	53
9	Accident on way to school	Child lives in urban area	3	90	40	1000	6.51	1	1		60
10	Bad posture in child	Heavy books carried daily	5	40	20	1000	9.52	1	0.5		60
11	Active feminist	Bank teller	5	0.4	2.11	100000	0.99				60
12	Blue cab	Eyewitness says “Blue”	15	80	20	100	41.38		0.5		60
13	Incorrect tax return	Error detected	20	30	10	1000	42.86	1	1	1	218
14	Choosing course in economics	Career-oriented	30	70	50	1000	37.5				60
15	Supplier A	Material defective	30	15	10	1000	39.13		1		221
16	Admission to school	Particular placement test result	36	75	20	1000	67.84				60
17	Get contract	Invited to second presentation	60	70	50	100	67.74		1	1	222
18	Produced in Ohio	Container Defective	60	5	10	1000	42.86		1		219
19	Red ball	Marked with star	80	75	25	500	92.31				60

*Tasks 13, 15, 17, and 18 have been taken from Hoffrage et al. (2015), and the others from Gigerenzer and Hoffrage (1995). For each task, two versions were constructed, one in which the information was presented in probabilities (Gigerenzer and Hoffrage, 1995) or percentages (Hoffrage et al., 2015) and one in which natural frequencies were used. The natural frequency versions can be constructed by applying the base rate to the size of the reference class. For instance, applying the base rate of 1% in Task 6 to the reference class of size 1,000 yields 10 out of 1,000. Applying the hit rate of 80% to these 10 yields 8 out of 10, and applying the false-alarm rate of 9.6 to the remaining 990 yields 95 out of 990. The three qualitative dimensions Norm Deviation (N), Stakes (S), and Main Focus (M) are explained in the text. The numbers of responses are aggregated across both task versions.*

Tasks 13, 15, 17, and 18 have been taken from Hoffrage et al. (2015; for the full descriptions, see their introduction, their Table 1, and their Appendix). The 440 participants who worked on these tasks were 259 undergraduate students of a business school and 181 managers in their role as students in an Executive MBA program. For each of the four tasks, two versions were constructed, one in which the information was presented in percentages and one in which natural frequencies were used. Each of the participants responded to two different tasks, either two percentage versions, or two natural frequency versions; in other words, representation format (henceforth the label for his variable) has been manipulated between-subjects.

Natural frequencies are the tallies in a natural sample in which hit rate and false-alarm rate are not normalized with respect to base rates (see Hoffrage et al., 2002 and Gigerenzer and Hoffrage, 2007; for an example of how probability information can be translated into natural frequencies, see the caption of **Table 1**). Natural Frequencies have proven to facilitate diagnostic inferences in laypeople (Gigerenzer and Hoffrage, 1995), advanced medical students and advanced law students (Hoffrage et al., 2000), patients (Garcia-Retamero and Hoffrage, 2013), physicians (Hoffrage and Gigerenzer, 1998), and managers and management students (Hoffrage et al., 2015). For a discussion about when and why natural frequencies are effective, see Gigerenzer and Hoffrage (2007), Brase (2008), Hill and Brase (2012), Brase and Hill (2015), and Johnson and Tubau (2015).

The remaining 15 tasks were taken from Gigerenzer and Hoffrage (1995; see Table 2, p. 293). In this study, four versions were constructed per task, but for the present re-analysis, we will only use two versions, namely the probability version and the natural frequency version of what Gigerenzer and Hoffrage (1995) called the standard menu. The information provided in the standard menu is displayed in **Table 1** (the two other versions involving the so-called short menu, which provides the information about the conjunctions *D*&*H* and *D*&–*H*, either in probabilities or in natural frequencies, are not included in the present re-analysis). Each of the 60 participants of Gigerenzer and Hoffrage (1995) received all 15 tasks in two versions. For 30 participants, these were probabilities, standard menu and natural frequencies, short menu, and for the other 30 participants, these were probabilities, short menu and natural frequencies, standard menu. The experiment took place in two sessions, most of them one week apart from each other. For each participant, half of the tasks in the first session were presented in one version, and the other half were presented in the other version. During a given session, a given participant has seen each task only once, that is, the two versions of the same task were given in different sessions. For the present re-analysis, which ignores all responses in the short menu version, this implies that we used a between-subject design: 30 participants responded to 15 tasks, each with information presented in terms of probabilities (seven tasks in one session and eight tasks in another session, one week apart from each other), and 30 other participants did the same, except that they were presented with the natural frequency versions of the same 15 tasks.

While both studies used a natural frequency condition, the condition with normalized information differed between the studies: Hoffrage et al. (2015) used percentages for their four tasks, and Gigerenzer and Hoffrage (1995) used probabilities for their 15 tasks. According to Gigerenzer and Hoffrage’s (1995) analysis (result 7, p. 689), this difference should not have an effect on Bayesian performance—a theoretical result that was confirmed by their own data and in numerous studies of other authors since then. We will thus pool the data from these two studies, and we will, henceforth, refer to this condition as the probability/percentage condition.

### Task Characteristics: Quantitative Dimensions

We will now introduce some candidate predictor variables that may explain some variance, across tasks, of participants’ responses. One obvious dimension along which the tasks vary is the numeric information: the base rate, the hit rate and the false-alarm rate. Note that the third example given in our introduction (Mellers and McGraw, 1999) was of this kind: the authors argued that the chance of responding with the Bayesian solution (which must not be confused with the Bayesian solution itself!) is affected by whether the base rate is high or low. There are some observations about this set of three quantitative variables that we can make already before looking at the participants’ data. First, the prior probability (i.e., base rate) is linked to the posterior probability: in our set of 19 tasks, the correlation is 0.76. The fact that this correlation is positive and substantial is trivial as the following analysis shows. Consider the so-called odds version of Bayes’ rule, which can be read as a division of two equations (more precisely: after the posterior odds ratio has been extended by *p(D)*/*p(D)*, the four numerators constitute one equation and the four denominators the other one):

(1) $$\underset{posterior\;odds\;ratio}{\underset{︸}{\frac{p(H|D)}{p\left(-H|D\right)}}}=\underset{prior\;odds\;ratio}{\underset{︸}{\frac{p(H)}{p(-H)}}}\times \underset{likelihood\;ratio}{\underset{︸}{\frac{p(D|H)}{p(D|-H)}}}$$

Equation 1 has the following implications: (a) if the likelihood ratio equals 1—which means that the data *D* is not at all diagnostic—then the posterior odds ratio is identical to the prior odds ratio, (b) if the likelihood ratio is larger than 1—which is usually the case and which is also the case for 17 of our 19 tasks—then the posterior odds ratio exceeds the prior odds ratio, and (c) the posterior odds ratio is a linear function of the prior odds ratio, with the likelihood ratio as a constant. Hence, one should expect a positive correlation between prior probability and posterior probability (although this link could be offset in a sample of tasks by some correlation patterns between the likelihood ratios and the corresponding prior odds in these tasks). For the sake of completeness, we want to mention that the correlation between base rate and the Bayesian solution (recall, 0.76) was found to be substantially higher than any other correlations that included the hit rate (Hr) and the false-alarm rate (F): corr (Bay^∗^Hr) = 0.16, corr (Bay^∗^F) = 0.34, corr (Br^∗^Hr) = -0.15, corr (Br^∗^F) = 0.51 and corr (Hr^∗^F) = 0.14.

### Task Characteristics: Qualitative Dimensions

Besides these quantitative dimensions, we categorized the 19 tasks along three qualitative dimensions. Note that the second example in our introduction (Krynski and Tenenbaum, 2007) was of this kind: these authors demonstrated that the chances of responding with the Bayesian solution is affected by whether or not a reason for the existence of false-alarms is given. We agree that this is an interesting variable and we embrace Johnson and Tubau’s (2015) problem solving approach to Bayesian reasoning that can account for why providing a reason facilitates Bayesian performance. We would have appreciated to also include this variable in the present analysis—yet, none of the 19 tasks provided such a reason, and accounting for variance on a criterion variable is pointless if there is no variance on the predictor variable. Fortunately, we were able to identify three other variables as meaningful and interesting for our purpose at hand.

The first variable is henceforth referred to as *norm deviation*. It denotes whether the focal hypothesis constitutes a deviation from a norm, in the sense that *H* can be considered an exception or something unusual that requires specific attention, whereas -*H* can be considered the normal case. To illustrate this variable with some examples from our set of 19 tasks, norm deviation has been coded as 1 for breast cancer (vs. non-breast cancer), HIV infection (vs. no infection), and incorrect tax report (vs. correct tax report). Note that such tasks can be conceived as signal-detection tasks: signals (or data, *D*) are used to detect norm deviations (or *H*). In contrast, norm deviation was coded as 0 for tasks in which it seemed to be hard, if not impossible, to say which was the normal case; for instance, red ball (vs. blue ball), blue cap (vs. green cap), or supplier A (vs. supplier B; for more examples, see **Table 1**).

Our second qualitative variable, henceforth referred to as *stakes*, denotes whether being in the state of *H* or -*H* makes a big difference (e.g., being infected with HIV, having an accident, or causing a prenatal damage has been coded as 1) or whether the stakes are either relatively low or not specified in the task description (e.g., drawing a red ball from an urn, being an active feminist, or choosing a course in economics has been coded as 0; **Table 1**).

Finally, our third qualitative variable is the *main focus* of the task. The main focus can either be on the individual case or on the numbers involved. For many tasks, the context story makes it clear that the central question is whether some individual or protagonist is in the state of *H* or -*H*, and the numbers given in the task description mainly serve the purpose of determining whether, given the observed data, this individual case should be treated as if *H* (vs. -*H*) were true. Examples include the questions of whether a specific woman (with a positive mammogram) has breast cancer or not, or whether a specific man (with fresh needle pricks) is a heroin addict or not. For such tasks, this variable has been coded as 1. In contrast, it was coded as 0 for tasks in which the main focus was on the relationship between the data *D* and the hypothesis *H*, in particular, on the posterior probability (or the corresponding relative frequency). The individual case is rather in the background and serves as an illustration. Examples include the Varden Soap task in which the vice president for production is not at all interested in the treatment of an individual soap container that was identified as defective, but in which he adopts a long run perspective and wonders about a fair allocation of costs between the two production facilities Ohio (*H*) and Virginia (-*H*) (see the appendix of Hoffrage et al., 2015).

### Dependent Variables

To find out how the quantitative and qualitative task characteristics can account for variance on participants’ responses, we analyze these responses in two ways. Specifically, our first dependent variable is the participants’ numerical estimate, which is continuous and comes in form of probabilities, percentages, or ratios, ranging from 0 to 100%. Our second dependent variable is the cognitive strategy a participant used to combine the given numbers (e.g., whether s/he provided the hit rate as a response). This is a categorical variable with as many levels as there are strategies, but it can also be seen as a vector of mutually exclusive binary (dummy) variables, each of which coded as present (i.e., with a value of 1) if a certain strategy is used, and which yields, aggregated across responses, proportions.

Two of these cognitive strategies that we used as a model in our analyses below are the base rate and the hit rate. The base rate is identical to the normative (i.e., the Bayesian) solution if the likelihood ratio is 1, that is, if the diagnostic information is not at all diagnostic—which is the case if the hit rate and the false-alarm rate are identical. Providing the hit rate as a response has been referred to as the “inverse fallacy” (Koehler, 1996; Villejoubert and Mandel, 2002), the “Fisherian algorithm” (Gigerenzer and Hoffrage, 1995) or the “conversion error” (Wolfe, 1995), and it has been accounted for by the representativeness heuristic (Kahneman and Tversky, 1972) or the “confusion hypothesis” (Macchi, 1995). Providing the false-alarm rate as a response happened in only 1.6% of the cases and so we decided to omit the results for this strategy in our analyses below (in Hoffrage et al., 2015, this occurred in 3.2% of the responses, see their Table 2; and it did not even pass the 1% threshold in Gigerenzer and Hoffrage, 1995, see their Table 3).

The two other cognitive strategies that we used are the Bayesian, as the normative response strategy, and the joint occurrence, which is the probability (or percentage) of cases in which both the data (*D*) are present and the hypothesis (*H*) is true: *p*(*D*&*H*). This number can easily be calculated by multiplying the base rate and the hit rate (*p(D*&*H*) = p(D| H)^∗^p(H); or by applying the hit rate information to the base rate of the focal hypotheses, e.g., 10 out of 1,000 women have breast cancer and 8 out 10 woman with breast cancer test positive, hence 8 out of 1,000 have breast cancer *and* test positive, see **Table 1**). Joint occurrence is the numerator of Bayes’ rule, and given that *p*(*H*|*D*) = *p*(*D*&*H*)/*p*(*D*), it can be seen as a step toward the Bayesian solution that falls short of carrying the computation to the end (see Johnson and Tubau, 2015). While we only classified responses as stemming from the base-rate or the hit-rate strategy when the responses were the exact values of the base rate or the hit rate, respectively, we used a more lenient criterion for those strategies for which the number could not simply be read off but had to be computed. Specifically, we classified responses as Bayesian or as stemming from the joint occurrence strategy when the responses were in the range of ±1% point from the value of the Bayesian solution or joint occurrence, respectively.

Gigerenzer and Hoffrage (1995) identified a wide range of other strategies, some of them were very exotic, have rarely been used, and basically reveal that participants had no clue and combined the numbers in an arbitrary and/or unreliable way. Such attempts come close to guessing, and many participants said right away that they simply guessed, without having been able to say in which way they used the numbers exactly. Whether ‘guessing’ deserves being labeled as a strategy is a matter of taste—pragmatically, that is, from a modeling point of view, it is useless as ‘guessing’ does not allow one to make predictions and to calculate goodness-of-fit measures. In sum, we restricted the report of our results to four cognitive strategies—Bayes, base rate, hit rate, joint occurrence—each of which made a precise point prediction. Based on the previous literature (in particular Gigerenzer and Hoffrage, 1995, and Hoffrage et al., 2015), these were the most frequently used strategies.

## Results

We structure the report of our results as follows: first, we analyze how the quantitative variables defined above are related to the qualitative dimensions of the tasks, and, second, how the qualitative variables are related to each other. Note that these analyses are conducted without any participant data. Third, we will present an overview of our data that comes close to presenting the raw data, thereby comprising all essential variables of the present analysis. Fourth, we will report how the quantitative and, fifth, how the qualitative task dimensions affect the numerical estimates. These analyses ignore process data and do not take into account whether a participant used a particular cognitive strategy, for instance, gave the hit rate as a response. Subsequently, we will turn to those 52.3% of the responses that have been identified as stemming from one of the most prominent strategies. For this subset of our data, we will analyze how, sixth, the representation format, seventh, the quantitative, and, eigth, the qualitative dimensions affect the usage of cognitive strategies.

### How are the Quantitative Dimensions Related to the Qualitative Dimensions?

In many real world contexts it may be the base rates that determine which category stands out and attracts special attention. In fact, for our 19 tasks, the correlation between base rate and norm deviation is -0.64 [average base rate for tasks with norm deviation coded as 1 and 0 is 3.4% and 35.1%, respectively, *t*(16) = 3.43, *p* = 0.004]. Similarly, the correlation between the Bayesian solution and norm deviation is -0.56 [average Bayesian solution for tasks with norm deviation coded as 1 and 0 is 11.9 and 44.1%, respectively, *t*(16) = 3.06, *p* = 0.008], that is, tasks for which *H* constitutes a norm deviation tend to have lower Bayesian solutions. Moreover, it can be expected that for these tasks (for which *H* can be seen as a norm deviation), the stakes are high. If we consider natural catastrophes, diseases, crimes, fraud, or failure of technical systems, then we find that these are not only rare events and norm deviations, but that there are usually also high incentives to detect them early in order to be able to intervene and to prevent the worst. In other words, stakes are high. Hence not surprisingly, Pleskac and Hertwig (2014) observed that for many events in the real world, probabilities and utilities are negatively correlated: the lower the probability of events, the higher their magnitude in utility terms, either as a cost (e.g., earthquakes with higher severities are less likely), or as a benefit (e.g., higher stakes lotteries are less likely to be won). Consistent with Pleskac and Hertwig (2014), the correlation between base rate and stakes that we observed in our set of 19 tasks is negative [-0.26; the average base rates for high stake tasks is 14.8% and for low stake tasks it is 30.2%; *t*(15) = 1.13, *p* = 0.27]. In turn, the Bayesian solutions for high stakes tasks are also lower (27.2%), than for low stakes tasks [41.2%, *t*(15) = 0.86, *p* = 0.40]. Also our third qualitative variable is correlated with some of the quantitative variables: even though the base rate for problems in which the main focus is on the individual is lower than when the main focus is on the numbers [11.9 vs. 22.1, *t*(17) = 0.86, *p* = 0.40], this does not translate into differences in the Bayesian solution [32.6 vs. 29.6, *t*(17) = -0.21, *p* = 0.83]. This pattern can be explained by a combination of both smaller false-alarm rates [10.1 vs. 17.9, *t*(17) = 1.0, *p* = 0.33] and higher hit rates [74.7 vs. 50.0, *t*(17) = -1.66, *p* = 0.12].

### How are the Qualitative Dimensions Related to Each Other?

All correlations in the triangle of qualitative variables are substantial and significant. The one between norm deviation and stakes is 0.62 (*p* = 0.005), that is, in tasks centering on norm deviations and abnormal cases, stakes tend to be high. The correlation between norm deviation and main focus is 0.45 (*p* = 0.05), that is, tasks about norm deviations tend to focus on the individual case. Moreover, the correlation between stakes and main focus is 0.55 (*p* = 0.01), that is, problems involving high stakes tend to focus on the individual case.

The results reported so far did not contain any participant responses and could hence have been reported before the first participant has shown up. Nevertheless, these are empirical findings that capture aspects of the statistical structure of Bayesian tasks. We will now turn to participants’ responses.

### How are Participants’ Responses Distributed in the 19 Tasks?

**FIGURE 1:**
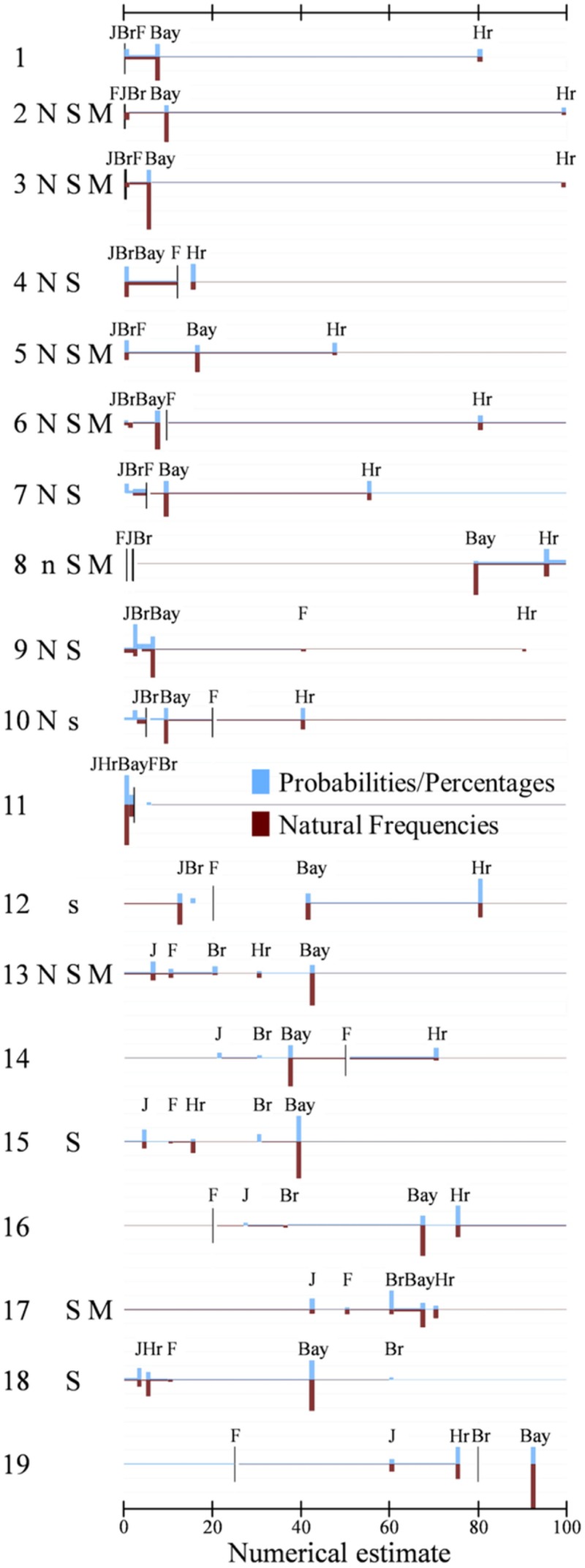
**Distribution of numerical estimates for the 19 tasks compiled in **Table 1** split by representation format**. The 19 tasks are displayed below each other, and sorted by their base rate. Responses to task versions in which numbers were presented in terms of probabilities or percentages are plotted, in blue color, above the *x*-axes, and responses to natural frequency versions are plotted, in red color, below the *x*-axes. The figure does not highlight all numerical estimates, but only those for the following five strategies: Bayes (Bay), base rate (Br), hit rate (Hr), false-alarm rate (F) and joint-occurrence (J). The *y*-axes of Figure plots the frequency of use of these strategies, that is, of giving the corresponding number as an estimate (one gridline indicates 20% points) against the number that correspond to these strategies (on the *x*-axes). These five strategies together account for a total of 53.9% of the responses; the remaining 46.1% are distributed across the six intervals that are defined by the five numerical estimates of the five strategies. The widths of the bars for the strategies was set to be 1% and the heights of the intervals between the strategies (or between the strategies and the end points of the scales) were chosen such that equal areas amount to equal percentages of participants responding with the corresponding numerical estimate (be it the precise number of a given strategy or any estimate that falls within a given interval). For some tasks, the numerical estimates corresponding to some strategies yielded a value between 0 and 1, so that our chosen resolution would require to plot them behind each other, with overlapping bars (and with the consequence that the total areas would no longer be constant across tasks). For these tasks, we stacked the areas corresponding to the involved strategies on top of each other, so that the bars gained in height and were comprised of different strategy users. Specifically, for Task 1 (probability version, *p*) the bar at 0 represents: 1 Br; Task 1 (natural frequency version, nf): 3J; for Task 4p: 4J and 2Bay; Task 4nf: 1J, 1Br, and 4Bay; Task 5nf: 1J and 2Br; Task 11p: 1J, 6Hr, and 5Bay; Task 11nf: 1J, 3Hr, and 13Bay. Moreover, for 10 of the tasks from Gigerenzer and Hoffrage (1999), a total of 46 responses (corresponding to 9.6% of all responses for these tasks) could not be displayed because they fell between two adjacent responses of different strategies that were too close to each other to allow for graphical representation. In addition, the figure contains the classification for the three qualitative variables introduced in section “Materials and Methods”: Norm deviation (N), Stakes (S), and Main focus (M). Capital letters denote that the hypothesized event constitutes a norm deviation, that stakes were high and that the main focus was on the individual case; absent letters denote the opposite; and letters in lower case denote that we could not agree how to code this variable (for instance, is being pregnant a norm deviation or not?). When computing the correlations or the regressions that are reported in the text, such unclear cases have been coded with 0.5, and when reporting the relative frequencies of strategy usage, the results for this variable level have been omitted.

**Figure 1** displays the 19 tasks listed in **Table 1**. It thereby uses the same order, namely the one established by the base rates, and the identification numbers in **Table 1** correspond to those in **Figure 1**. This figure comes close to a presentation of the raw data. It visualizes, for each task, all variables that are included in the present analyses: the two sets of predictor variables (quantitative and qualitative task dimensions), and the two kinds of dependent variables (numerical estimates and response strategies). The quantitative dimensions of the task are included as lines that represent the numerical values of the base rate (Br), of the hit rate (Hr), of the false-alarm rate (F), and also of the Bayesian solution (Bay). The letters that stand for the three qualitative dimensions introduced above—norm deviation (N), stakes (S), and main focus (M)—indicate that the corresponding variable has been coded as “1” (absence of a letter for a given task indicates that the dimension has been coded as “0”). On the side of the dependent variables, the figure displays the distribution of numerical estimates, highlighting the estimates that correspond to specific response strategies in vertical bars, while all other responses that could not be assigned to one of the strategies that we selected for this analysis are visualized in a horizontal bar. The height of the vertical bars depicts the relative frequency of response strategy usage.

The advantage of this kind of data representation is, at the same time, its disadvantage. The figure contains a lot of information and is very detailed. In the subsequent sections we will hence focus on specific effects that the predictor variables exert on the dependent variables, that is, we split the data into subgroups and aggregate them so that some effects become better visible.

### How do the Quantitative Dimensions Affect the Numerical Estimates?

**FIGURE 2:**
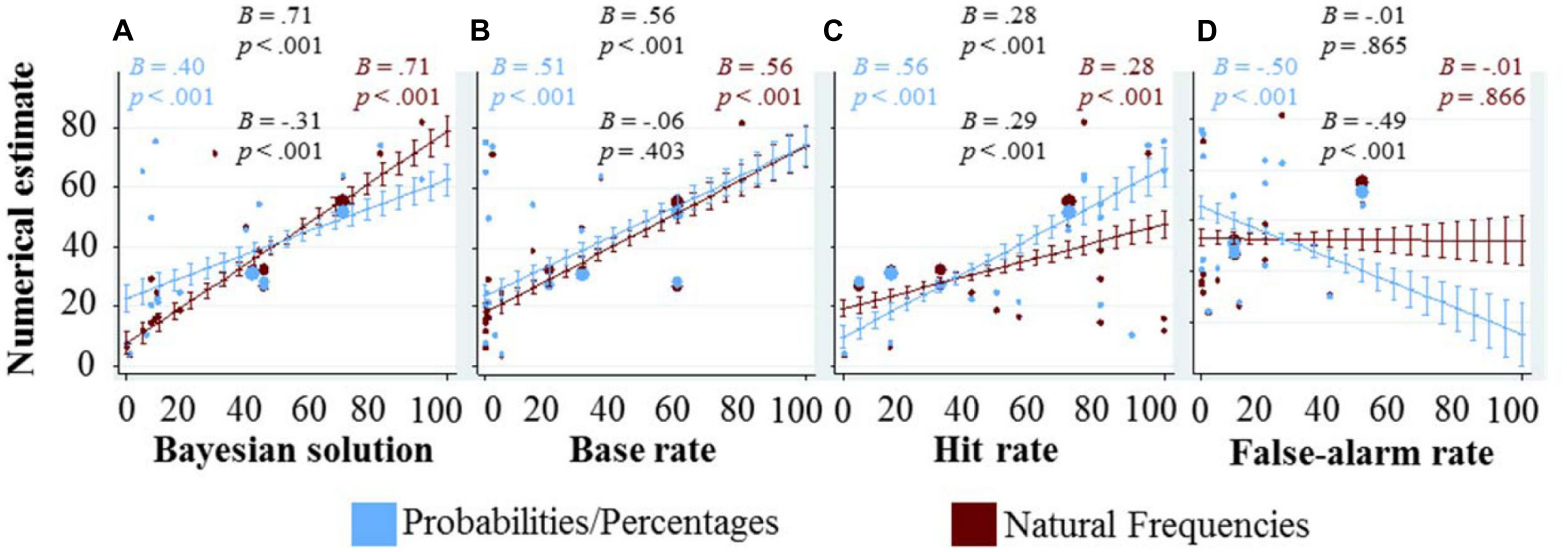
**Graphical exploration of how the quantitative variables (displayed at the x-axes) affect the numerical estimates (displayed at the y-axis).** Each blue dot represents the average response in the probability/percentage version of a task, each red dot the average response in the natural frequency version, and the size of each dot indicates the number of responses on which these averages are based (see also the rightmost column of **Table 1**). The marginal effects and their confidence intervals are based on regression analysis. In **(A)** showing the Bayesian solution on the *x*-axis, the regression included only representation format and the Bayesian solution as main effects, as well as their interaction. It does not include the three quantitative variables as control variables, as the Bayesian solution already combines them (according to Bayes’ rule). The other three panels **(B–D)** display the three quantitative variables, base rate, hit rate, and false-alarm rate on the *x*-axis. Each of the marginal effects and confidence intervals was computed with representation format and all three quantitative variables as main effects as well as interaction effects between representation format and each of the three quantitative variables. In all regressions, standard errors were clustered for each participant. We included four coefficients and corresponding *p*-values in each panel. If at least one of the four *p*-values in a given panel was lower than 0.1, then all four coefficients and their corresponding *p*-values are displayed; otherwise not a single number is reported. The reported numbers are arranged in the shape of a diamond, the main effect on top and the interaction with representation format at the bottom. On the left, we included the main effect when calculating the regression only for responses in the probability/percentage condition, and on the right the main effect in the natural frequency condition. It does not come as a surprise that the numbers on top (main effect) and the numbers on the right (main effect in the natural frequency condition) are almost identical: the interaction is coded as the interaction with the probability/percentage condition, thus the main effect captures the effect in the natural frequency condition. The number on the left can thus also be calculated by adding the main effect (the numbers on top) and the interaction (numbers on the bottom).

To see how the numbers given in the task affect the numerical estimates of the participants, we choose a data representation that combines (a) scatter-plots in which each dot denotes the average numerical estimate for a given task and information representation format, with (b) marginal effects from regression analysis and their corresponding confidence intervals (**Figure 2**).

**Figure 2A** shows the numerical estimates as a function of the Bayesian solution. If every participant would have given the Bayesian response, the slope would have been one. Both slopes fitting the participants’ estimates are smaller than 1, but the slope in the frequency condition is significantly steeper than in the percentage condition. This is partly due to the fact that the proportion of Bayesian responses was higher in the frequency condition; however, this interaction effect also persists when looking only at the non-Bayesian responses (*B* = -0.21, *p* < 0.001). In particular, the slope in the probability/percentage condition decreased from *B* = 0.40 when all responses were taken into account to *B* = 0.30 when considering only the non-Bayesian responses, and the slope in the frequency condition decreased from *B* = 0.71 to *B* = 0.51 (all *p*’s < 0.001). Moreover, we found that participants that were presented information in terms of natural frequencies were more likely to respond with the Bayesian solution; and if they did not, their responses were on average closer to the Bayesian solution [note that this decrease in average absolute differences among the non-Bayesian responses could not be observed for the subset of the four tasks taken from Hoffrage et al. (2015)—to the contrary, there we even found the opposite].

**Figure 2B** shows that, when statistically controlling for the other two quantitative variables, higher base rates lead to higher numerical estimates. As we have discussed above when introducing the odds version of Bayes’ theorem, the prior odds, represented by the base rate, should be positively correlated to the posterior odds. Not surprisingly, such a positive correlation could also be observed between base rates and participants’ numerical estimates of posterior probabilities. Again, this effect is partly driven by participants who give the Bayesian response, however, it persists even after all Bayesian responses have been excluded from the regression; in fact, this exclusion reduced the coefficient (*B* = 0.31) but it still remains significant (*p* < 0.001). At the same time, higher base rates are also associated with higher absolute differences between numerical estimates and Bayesian solutions (overall *B* = 0.09, *p* = 0.016; and when only considering the non-Bayesian responses *B* = 0.25, *p* < 0.001). In sum, while more participants find the Bayesian solution for tasks with higher base rates, those participants who do not find the Bayesian solution make larger mistakes in these tasks.

Similarly to the base rate, the hit rate also has a positive effect on the numerical estimate, as can be seen **Figure 2C**. As for the other analyses (**Figures 2A,B**), this effect also persists after all Bayesian responses have been excluded from the regression analysis (*B* = 0.35, *p* < 0.001). Interestingly, in the frequency condition the influence of the hit rate on the numerical estimate is significantly weaker. Additional analyses reveal that only in the probability/percentage condition, higher hit rates are associated with higher absolute deviations from the Bayesian response (*B* = 0.30, *p* < 0.001), and that this effect persists after excluding all Bayesian responses from the analysis (*B* = 0.28, *p* < 0.001).

In **Figure 2D**, it can be observed that the false-alarm rate is strongly negatively related to the numerical estimate in the percentage condition, but unrelated in the frequency condition. The partial correlation between the false-alarm rate and the Bayesian solution (after statistically controlling for the base rate and the hit rate) is -0.13, implying that participants in the probability/percentage condition are overreacting to the false-alarm rate, and participants in the natural frequency condition are not reacting enough. Note that in none of the 19 problems, the false-alarm rate was above 50%, and thus the marginal effects estimates for this area are based on pure extrapolation.

In sum, the three numbers provided in the task are related to the Bayesian solution and Bayes’ rule quantifies how the exact relationships are. Generally speaking, the higher the base rate, the higher the Bayesian solution; the higher the hit rate, the higher the Bayesian solution; and the higher the false-alarm rate, the lower the Bayesian solution. Each of these three relationships could be found for the numerical estimates as well. Interestingly, they could also be found even among the non-Bayesian responses. When establishing these relationships for one of the three quantitative dimensions through regression analyses, we controlled for the other two. Note that this statistical control has its limits, because a regression can only do so through a linear combination, while Bayes’ rule is not a simple linear combination. In a way, Bayes’ rule is the normative correct way how to take all three pieces of information into account, and this is exactly what we have done in **Figure 2A**—which nicely shows that numerical estimates could very well be predicted through the three numbers provided in the task.

### How are the Qualitative Variables Related to the Numerical Estimates?

**FIGURE 3:**
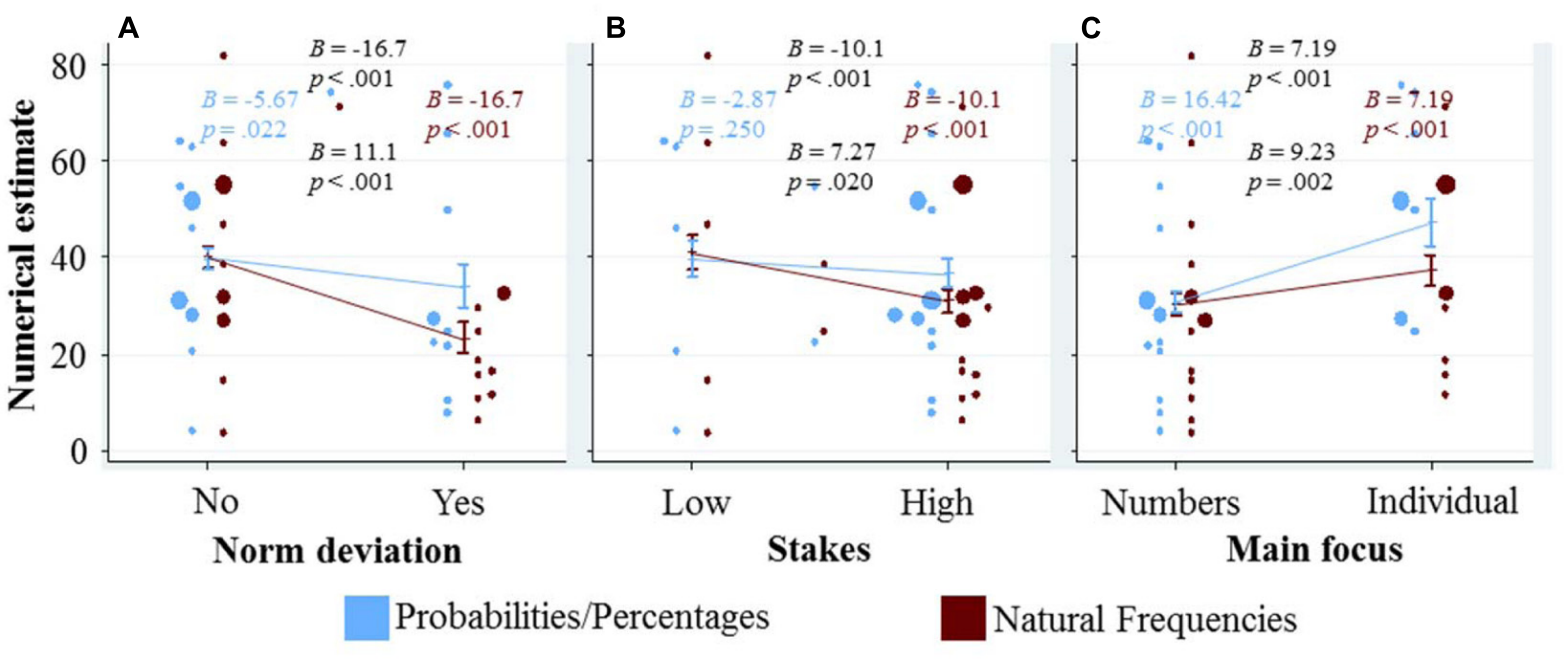
**Graphical exploration of how the qualitative variables (displayed at the x-axes) affect the numerical estimates (displayed at the y-axis).** To avoid overlap of the dots (see the caption of **Figure 2** for details what they represent), the blue dots are displayed slightly to the left of the confidence interval for the marginal effect, and the red dots slightly to the right. In addition, blue (red) dots that would overlap with other blue (red) dots are moved slightly further to the left (right). The marginal effects and their confidence intervals for each of the qualitative variables **(A–C)** are calculated in a separate regression that only includes the respective qualitative variable, representation format and their interaction (SE were clustered for each participant). We included the four resulting coefficients and corresponding *p*-values in each panel (see caption of **Figure 2** for details).

To see how the three qualitative variables characterizing a given task affect the numerical estimates of the participants, we adapted the data representation of our previous question as follows: in **Figure 3**, the three qualitative variables are depicted on the respective *x*-axis of the three panels, and the numerical estimates are plotted on the *y*-axis. As in our previous figure, we again display a dot for the mean numerical estimate of each task in each representation format conditions, and combine this with marginal effects and their confidence interval from regression analysis. The marginal effects of each of the qualitative variables are calculated in a separate regression that only includes the respective qualitative variable, representation format and their interaction. We used separate regressions to explore the differences in responses between tasks, as if the qualitative variable was the only dimension on which the tasks differed. Thus, all the differences between the tasks with a specific quality (e.g., norm deviation = 1) and the tasks without that quality (e.g., norm deviation = 0) will be reflected in the marginal effect shown in **Figure 3**. These marginal effects can thus be seen as an upper bound of the effect of the qualitative variable (unless these qualitative variables are confounded with others factors that have an opposing effect, if this were the case, then ‘controlling’ for these other factors will increase the observed effects).

**Figure 3A** shows that the numerical estimates are lower for norm deviation tasks. This negative effect is (significantly) larger within the frequency condition compared to the probability/percentage condition. This pattern can party be explained by the lower Bayesian solutions for the problems where norm deviation is 1, and by the larger number of Bayesian responses in the frequency condition. However, even when excluding all Bayesian responses, both the main effect (*B* = -10.88, *p* < 0.001) and the interaction (*B* = 8.99, *p* = 0.007) remain significant.

**Figure 3B** depicts a similar, yet less pronounced pattern for the variable stakes. Like for norm deviation, this pattern is partly, but not only, driven by differences in the percentage of Bayesian responses (when only considering the non-Bayesian responses: *B* = -10.3, *p* = 0.002 for the main effect and *B* = 8.3, *p* = 0.065 for the interaction with representation format).

**Figure 3C** visualizes the effect of the variable main focus. Participants gave significantly higher numerical estimates for tasks in which the main focus was on the individual case, compared to tasks where the main focus was on the numbers. In contrast to the effects depicted in the other two panels, the effect of main focus is significantly more pronounced in the probability/percentage condition than in the frequency condition. This is particularly interesting, as the Bayesian solutions seem to be unaffected by this variable (*r* = 0.05), and thus a main focus on the individual seems to distract participants from finding the Bayesian solution (and this distraction effect is stronger in the probability/percentage condition).

### How does Representation Format Affect the Usage of Cognitive Strategies?

In the previous sections we focused, unless otherwise noted, on all responses and took the numerical estimates as the dependent variable. We will now restrict the analyses only to those responses that have been categorized as Bayesian, or that were identical to either the base rate provided in the task, the hit rate, or the joint occurrence of *D* and *H*. Across both representation formats, any of these four strategies was used in 52.3% of our 1,773 responses. The most frequent strategy, across both formats, was the Bayesian strategy (with 27.7%). The second most often used strategy was the hit rate, but with 11.1% it was used far less often than in other studies (e.g., Villejoubert and Mandel, 2002). The third and fourth most often used strategies were joint occurrence (with 9.2%) and base rate (with 4.3%), respectively.

How did strategy use depend on format? Averaged across all participants and all 19 tasks, the Bayesian strategy was used in 16.9% of cases for probability/percentage representations, and in 38.5% of cases for natural frequency representations (*p* < 0.001, in a logistic regression with standard errors clustered for each participant). For the base rates, these numbers were 6.4 and 2.3%, respectively (*p* < 0.001), and for joint occurrence, 12.1 and 6.2%, respectively (*p* < 0.001). In contrast, format did not exert a significant effect on responding with the hit rate (10.4 vs. 11.7%, respectively; *p* = 0.61) and also not on the usage of the false-alarm rate (1.2 vs. 2%, respectively; *p* = 0.23; the false-alarm rate is not displayed in the Figures and will no longer be considered in the analyses below).

### How do the Quantitative Dimensions Affect the Usage of Cognitive Strategies?

**FIGURE 4:**
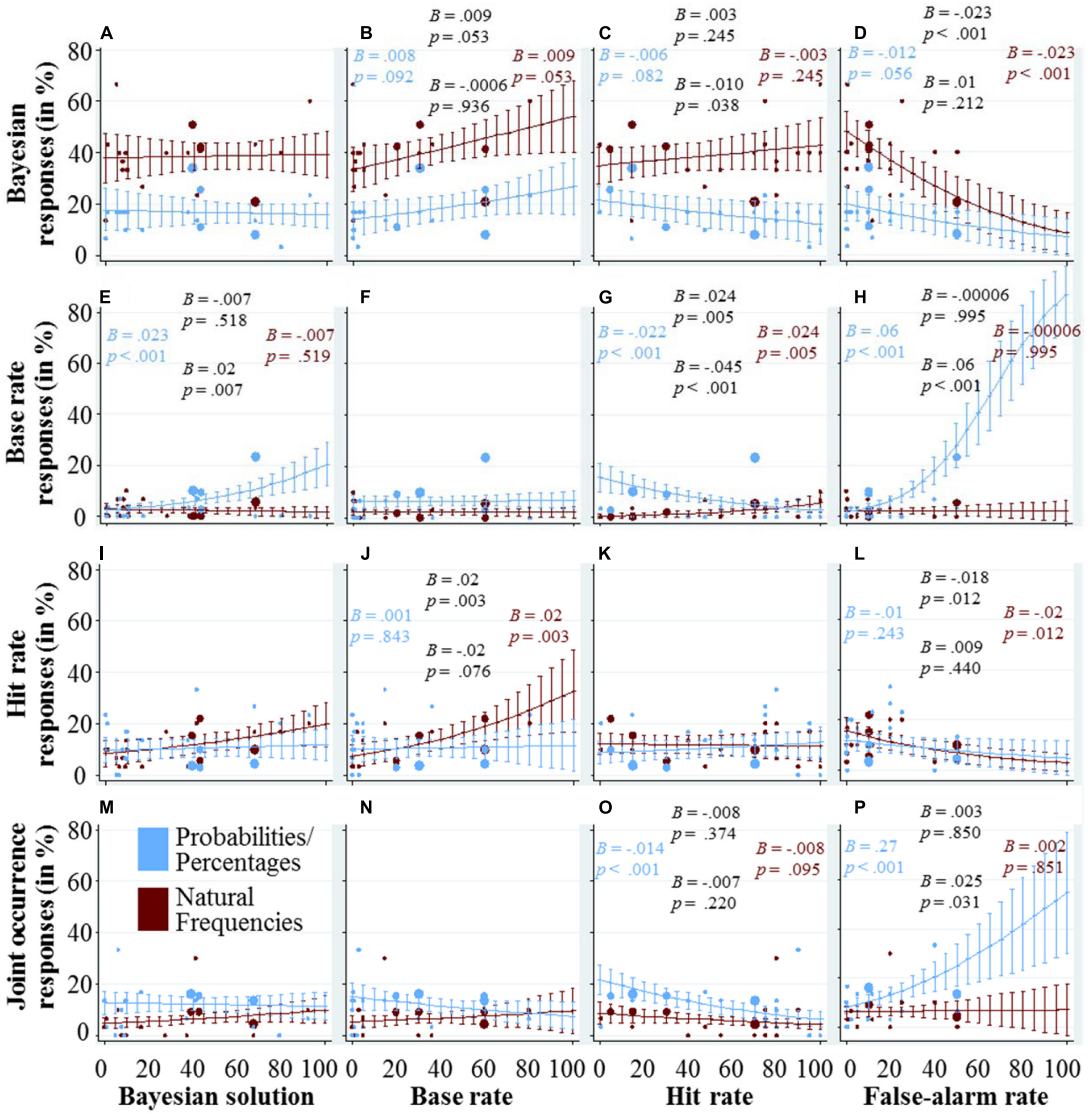
**Each of the panels (A–P) visualizes how the quantitative variable that is displayed at the x-axis affects the usage of the cognitive strategy that is displayed at the y-axis.** The dots represent the average usage in the different tasks and versions (see caption of **Figure 2** for details). The marginal effects and their confidence intervals are based on logistic regression analysis with strategy use as the dependent variable, following the same specifications as the regression analyses used for **Figure 2** (see the caption of **Figure 2** for details). We also included the four coefficients and corresponding *p*-values in each panel (again, see the caption of **Figure 2** for details).

As in the last figures, **Figure 4** combines scatter-plots to represent the different tasks in both representation format conditions, with marginal effects and their confidence intervals from regression analysis. In the panels depicted in the first row (**Figures 4A–D**), we explore how the quantitative variables influence participants’ performance in finding the Bayesian solution. **Figure 4A** shows that the percentage of participants responding with the Bayesian solutions does not depend on what the Bayesian solution is. In **Figure 4B**, there is a trend indicating that the higher the base rate, the more participants find the Bayesian solution. The effect of the hit rate, depicted in **Figure 4C**, depends on the representation format. In the probability/percentage condition, higher hit rates seem to lead to less Bayesian responses, whereas in the frequency condition, the effect of the hit rate seems to be smaller and in the opposite direction. A potential explanation can be found in the other panels of the third column. When the hit rate is low, participants in the probability/percentage condition used the base rate and the joint occurrence more often as a response strategy. In the last panel of the first row (**Figure 4D**), it can be seen that the higher the false-alarm rate, the smaller the percentage of participants who found the Bayesian solution. In the probability/percentage condition, this can again be partly explained by a higher reliance on the hit rate and joint occurrence as a response strategy. In the frequency condition, however, it is unclear which strategy those participants used who failed to find the Bayesian solution. Note that in the 19 tasks we investigated, the highest false-alarm rate was at 50%, which makes the estimates in the right part of the panel based on pure extrapolation.

### How do the Qualitative Dimensions Affect the Usage of Cognitive Strategies?

**FIGURE 5:**
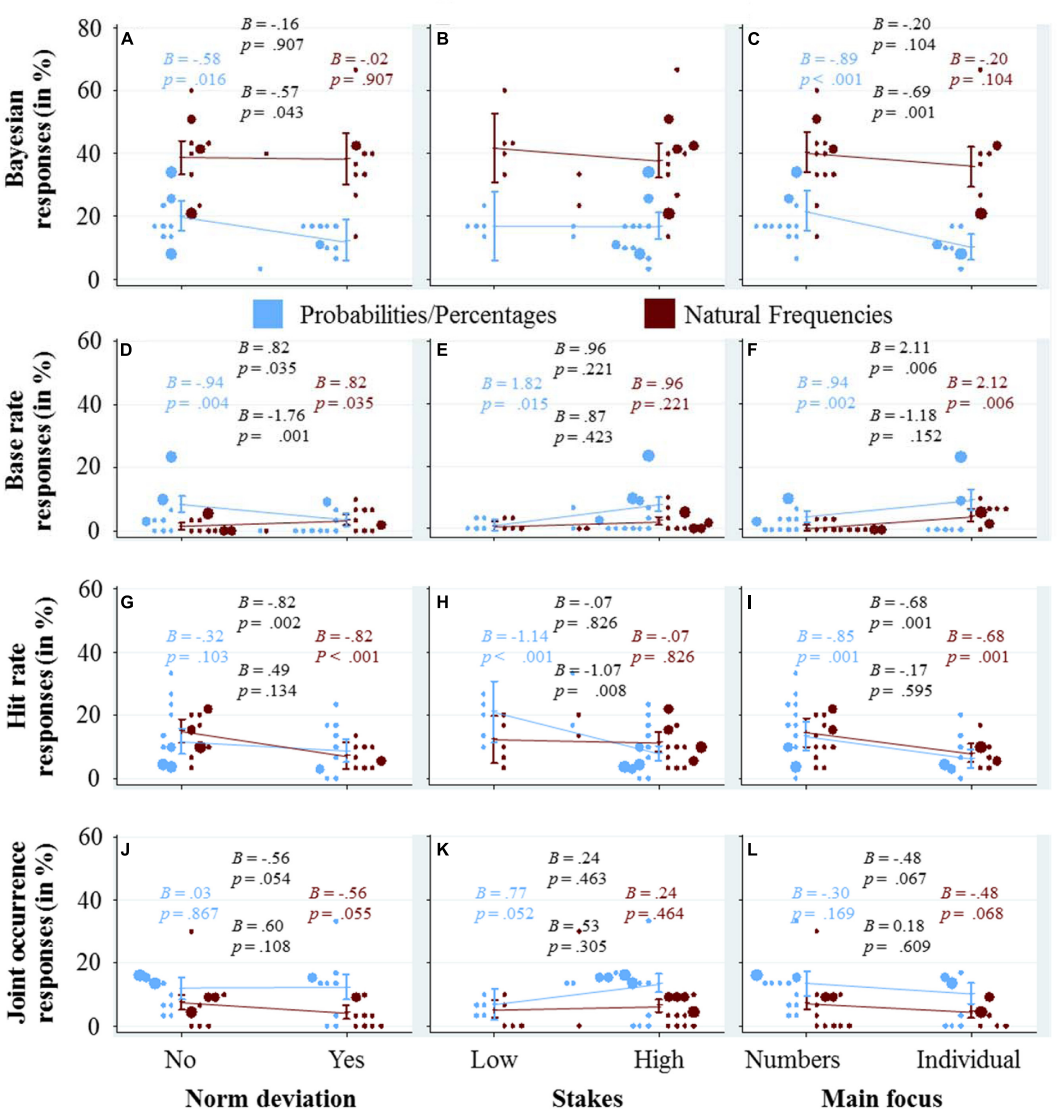
**Each of the panels (A–L) visualizes how the qualitative variable that is displayed at the x-axis affects the usage of the cognitive strategy that is displayed at the y-axis.** The dots represent the average usage in the different tasks and versions (see caption of **Figure 2** for details about their color and size). The marginal effects and their confidence intervals are based on logistic regression analysis with strategy use as the dependent variable, following the same specifications as the regression analyses used for **Figure 3** (see the caption of this figure for details). We included four coefficients and corresponding *p*-values in each panel (see also the caption of **Figure 2** for details).

In **Figure 5**, we explore graphically the effect of the three qualitative variables, norm deviation, stakes and main focus (in the three columns) on the response strategies (in the four rows), again using a combination of scatter-plots and marginal effects with confidence intervals. For the marginal effects and their confidence intervals, we calculated a separate logistic regression for each panel because we did not want to explore the unique contribution of the quantitative variables, but rather the upper bound of their explanatory power, under the assumption that they represent the only difference between the tasks (as in **Figure 3**).

The panels in the first row depict the effects of the qualitative variables on the percentage of Bayesian responses. **Figure 5A** shows that in the probability/percentage condition, it is harder for participants to find the Bayesian solution for tasks with norm deviation, while in the frequency condition the percentage of Bayesian responses did not seem to depend on whether a task includes a norm deviation or not. In **Figure 5B**, it can be seen that whether a task has high or low stakes does not significantly affect the percentage of Bayesian responses. For the sake of completeness, let us mention that when the stakes are high (compared to low), participants in the probability/percentage condition seem to respond more often with the base rate (**Figure 5E**), less often with the hit rate (**Figure 5H**), and more often with the joint occurrence (**Figure 5K**)—while participants in the frequency condition remain largely unaffected by the stakes. A main focus on the individual seems to negatively affect participants’ performance in finding the Bayesian solution, especially in the percentage condition (**Figure 5C**). Instead, slightly more participants used the base rate as a response (**Figure 5F**).

## Discussion

In this paper we explored the effects of three quantitative and three qualitative dimensions characterizing Bayesian inference tasks on participants’ responses. To accomplish this, we plotted the responses of 500 participants to 19 different tasks in several ways. We started broadly with the numerical estimates participants provided as responses, and afterwards classified some of their responses as stemming from different response strategies. We differentiated the tasks both based on the quantitative variables that define the statistical problem—namely, the base rate, hit rate, and false-alarm rate—and on qualitative variables that describe the context and narrative of the task. In this explorative analysis, we found that participants seem not to perceive all Bayesian inference tasks as being equal, and most of the variables we investigated seem to influence not only the specific numeric response participants are providing, but—and of course not independently of the numeric responses—which strategy they use. In the remainder of this paper, we want to highlight three main lessons we draw from this exploratory investigation, and we outline some avenues for future research.

First, the numerical value of the Bayesian solution does not seem to influence whether participants find it. While their responses are driven by the different pieces of information stated in the task (base rate, hit rate, and false-alarm rate), and by other qualitative variables that can be seen as irrelevant from a normative point of view, their response strategy seems to be unaffected by what the Bayesian solution is. For our set of 19 tasks, about the same proportion of individuals provides the Bayesian solution, independent of whether it is as low as 0.03% or as high as 92.3%. However, the large majority of the participants’ responses (77.9%) were from a task for which the Bayesian solution was 42.9% or less.

Second, focusing on the numbers, instead of the individual case, seems to increase participants’ performance. Interestingly, this effect was more pronounced in the probability/percentage condition and less pronounced in the natural frequency condition. To better understand the effect of main focus, it is useful to consider the debate about the underlying mechanism of the beneficial effect of natural frequencies (Gigerenzer and Hoffrage, 2007; Brase, 2008; Hill and Brase, 2012; Brase and Hill, 2015; Johnson and Tubau, 2015). The two prominent explanations for the beneficial effect of natural frequencies are that they (a) make the nested set relationship more explicit, and that they (b) prompt participants to think in terms of frequencies (instead of “single event probabilities”). Brase (2008) provided evidence for the second explanation: participants who interpreted the somewhat ambiguous word “chances” as frequencies performed better than those participants who interpreted “chances” as probabilities. In line with this explanation, a main focus on the numbers might lead participants to adopt a frequentist point of view, thereby increasing their performance. In contrast, a main focus on the individual case might prevent participants from adopting and, in turn, benefiting from such a viewpoint. This account would also explain why the effect of main focus was more pronounced for probability representations, where Bayesian performance tends to be low and thus leaves more room for the effect of focusing on the numbers—while for natural frequency representations a main focus on the numbers had less added value as most participants were already thinking in terms of numbers anyway (but the effect of main focus could still be observed even within the frequency condition, see **Figure 5C**).

The practical consequences of the main focus might be particularly severe, as the tasks with a focus on the individual case tend to be about a norm deviation and tend to have high stakes, at least in our sample of tasks. Of course we cannot make any causal claims here, but our results are consistent with the following speculation: if a specific problem involves a norm deviation and if stakes are high, those who formulate a problem may be led to focus on the individual case, for instance, to attract the readers’ attention, to appeal to emotions, and to increase empathy (cf. the identified-victim effect, Small and Loewenstein, 2003). They may even adopt such a focus with good intentions, namely to increase the readers’ involvement and motivation to solve the problem. And even if a task description is relatively neutral, chances are that the reader may focus on the individual if the hypothesis involves a norm deviation and if stakes are high. However, and ironically, such a frame increases the difficulty of the problem, as our results suggest, and may more than offset any beneficial effect that the increased motivation and the personal affection might have. It may sound trivial, but this points to a potential strategy how problems could be reframed (or how individuals could reframe them in their head) to boost the accuracy of responses: use natural frequencies rather than probabilities to communicate the statistical information, and, on top of this, focus on the numbers rather than on the individual case. However, as our analysis is only exploratory, future research would be needed to systematically test such a reframing strategy, and to disentangle the effect of ‘main focus’ of the task from other effects and to identify potential boundary conditions.

Third, in the probability/percentage condition, the quantitative and qualitative task characteristics influenced participants’ responses to a larger extent than in the natural frequency condition. This could possibly be explained by the fact that the percentage of Bayesian responses was higher in the natural frequency condition (on average there were 38.5% Bayesian responses in the natural frequency condition and only 16.9% in the probability/percentage condition). For someone who figured out how to structurally solve the Bayesian inference tasks (Johnson and Tubau, 2015), there was no need to find a solution in the particulars of the task specific context stories or to use a non-Bayesian strategy, for instance, by taking one of the numbers provided in the task or by integrating them in some other way. In contrast, someone who did not understand how the numbers should be combined could be tempted to look for similarities between the problem at hand, and problems they have solved before. In other words, for someone who figured out what the normative response strategy is, the task content and any other characteristics were exchangeable decoration—and for those who did not, such variables could possibly exert an influence.

Yet, in the natural frequencies condition, many participants also struggled with the tasks and where hence vulnerable to task dimensions that are irrelevant from a normative point of view. Why are Bayesian tasks still hard for some, even when information is presented in terms of natural frequencies? One reason could be that outside of the lab, most situations in which individuals update their beliefs do not feature numerical information about base rates, hit rates, and false-alarm rates. For some participants, it might have been the first time that they encountered such text book problems when they read the descriptions of the tasks in the context of the experiment. Outside of the lab, information updating might often rather consist of evaluating some data/sampling some information, based on which individuals form their initial beliefs, and then afterward evaluating some more data, maybe more locally relevant or more recent, and then revising their beliefs in light on the new data. Of course, in their statistical structure, such situations are different from Bayesian inference tasks, but because individuals have much more experience with other information updating tasks, they might try to rely on this experience to make sense of the Bayesian inference tasks. And, outside of the environment of Bayesian inference tasks, information updating strategies that are contingent on task specific factors such as the trustworthiness of the initial data or the new data (Welsh and Navarro, 2012) or the judgment of the validity of the sample (Fiedler, 2000) might be ecologically rational.

Overall, we can conclude from these exploratory analyses that not only the quantitative variables (the numbers given in the task) but also our qualitative variables (norm deviation, stakes and main focus) could explain some variance in participants’ responses and in particular in the strategies they use. However, even though most of the effects of our six predictor variables on the four strategies that we inspected reached statistical significance, we hasten to add that such a result is not too hard to achieve with 1,773 responses and that most of the differences between the percentage points of strategy use, contingent on the levels of our dichotomous predictor variables, was in the order of five percentage points. Given that most percentages were close to the lower end of the scale, such differences are, relatively speaking, quite large, but with respect to the whole scale of 100%, a difference of 5% points is still a small difference. Moreover, it is important to consider that this analysis is based on a *post hoc* analysis of only 19 tasks, and that these tasks were not designed to allow for systematic tests of the quantitative and qualitative dimensions. For instance, as norm deviation is highly correlated with the base rate and the Bayesian solution (and not orthogonally manipulated), it is not possible to causally attribute the observed effect to either the qualitative dimension (i.e., the norm deviation narrative) or the underlying quantitative dimensions (i.e., the numbers).

One avenue for future research could hence be to use constructed scenarios and manipulate some of the variables used in the present analysis systematically, that is, orthogonally. A prime example for this approach is Krynski and Tenenbaum’s (2007) study that we mentioned in Section “Introduction”: these authors manipulated one aspect of the task while keeping everything else constant. This would naturally allow for conclusions that have a much higher internal validity, compared to the observations we can share and the tentative conclusions we can formulate based on our exploratory analyses, which were based on a comparison between tasks that differed on many aspects simultaneously.

Another avenue would be to go in the opposite direction: not to use systematic designs, but what Brunswik called a representative design (see Dhami et al., 2004). Even though we referred to the present analyses as a first step toward an ecological analysis of Bayesian inferences, we must acknowledge that it does not fully deserve this label. For many of the 19 tasks, the base rates and the statistical properties of the diagnostic test have been made up rather than measured in a real-world context. It would hence be interesting to conduct such an analysis and to study the dimensions that may affect strategy use in larger pool of Bayesian inference tasks from real-world applications and with natural inter-correlations between the variables of interest.

For many study participants, Bayesian inference tasks are hard, and most responses are not Bayesian. Moreover, the qualitative task characteristics that we scrutinized in our analyses should not play a role from a normative point of view, however, they did influence participants’ responses and they also had an impact on which cognitive strategy they used. How can one account for non-normative responses and for the finding that task characteristics that should be irrelevant from a normative point of view *did* play a role? A promising approach to answer this question may involve making an attempt to put oneself into participants’ shoes and to ask how they approach the task. Which mental models (Gentner and Stevens, 1983; Johnson-Laird, 1983) do they construct? What is their problem space (Simon and Newell, 1971; Newell and Simon, 1972)? What kinds of belief updating tasks do they encounter in their environments and how could their experience with these tasks possibly inform solutions to this special class of belief updating tasks that come in the form of textbook problem? As researchers who study how participants change their beliefs in light of new data, we may eventually find out that we may need to change our perspective, research questions, and research paradigms in light of new experimental findings. Adopting the perspective of individuals who have to solve Bayesian tasks, and aiming at understanding what constitutes the environment of comparable tasks from their perspective seems to be a fruitful avenue for future research.

## Conflict of Interest Statement

The authors declare that the research was conducted in the absence of any commercial or financial relationships that could be construed as a potential conflict of interest.
